# Cost-effectiveness of HIV care coordination scale-up among persons at high risk for sub-optimal HIV care outcomes

**DOI:** 10.1371/journal.pone.0215965

**Published:** 2019-04-25

**Authors:** Elizabeth R. Stevens, Kimberly A. Nucifora, Mary K. Irvine, Katherine Penrose, McKaylee Robertson, Sarah Kulkarni, Rebekkah Robbins, Bisrat Abraham, Denis Nash, R. Scott Braithwaite

**Affiliations:** 1 Department of Population Health, NYU School of Medicine, New York, NY, United States of America; 2 Bureau of HIV/AIDS Prevention & Control, New York City Department of Health and Mental Hygiene, New York, NY, United States of America; 3 Institute for Implementation Science in Population Health, City University of New York, New York, NY, United States of America; 4 Department of Epidemiology and Biostatistics, School of Public Health, City University of New York, New York, NY, United States of America; AIDS Healthcare Foundation, UNITED STATES

## Abstract

**Background:**

A study of a comprehensive HIV Care Coordination Program (CCP) showed effectiveness in increasing viral load suppression (VLS) among PLWH in New York City (NYC). We evaluated the cost-effectiveness of a scale-up of the CCP in NYC.

**Methods:**

We incorporated observed effects and costs of the CCP into a computer simulation of HIV in NYC, comparing strategy scale-up with no implementation. The simulation combined a deterministic compartmental model of HIV transmission with a stochastic microsimulation of HIV progression, and was calibrated to NYC HIV epidemiological data from 1997 to 2009. We assessed incremental cost-effectiveness from a health sector perspective using 2017 $US, a 20-year time horizon, and a 3% annual discount rate. We explored two scenarios: (1) two-year average enrollment and (2) continuous enrollment.

**Results:**

In scenario 1, scale-up resulted in a cost-per-infection-averted of $898,104 and a cost-per-QALY-gained of $423,721. In sensitivity analyses, scale-up achieved cost-effectiveness if effectiveness increased from RR1.11 to RR1.37 or costs decreased by 41.7%. Limiting the intervention to persons with unsuppressed viral load prior to enrollment (RR1.32) attenuated the cost reduction necessary to 11.5%. In scenario 2, scale-up resulted in a cost-per-infection-averted of $705,171 and cost-per-QALY-gained of $720,970. In sensitivity analyses, scale-up achieved cost-effectiveness if effectiveness increased from RR1.11 to RR1.46 or program costs decreased by 71.3%. Limiting the intervention to persons with unsuppressed viral load attenuated the cost reduction necessary to 38.7%.

**Conclusion:**

Cost-effective CCP scale-up would require reduced costs and/or focused enrollment within NYC, but may be more readily achieved in cities with lower background VLS levels.

## Introduction

Treatment advances have improved health and survival for persons living with HIV (PLWH), as well as opportunities to prevent transmission.[1–4] However, along the care continuum there are many challenges to maximizing the individual and public health benefits of treatment,[5–11] and outcomes remain persistently suboptimal throughout the US, with 15% of the estimated 1.11 million PLWH being undiagnosed and only 57.9% experiencing viral load suppression within six months of diagnosis (VLS).[12, 13] Although achieving better care continuum outcomes than other large US cities, New York City (NYC) has to make further progress on VLS in order to meet UNAIDS 90-90-90 targets.[14] In 2016, of an estimated 87,700 PLWH in New York City (NYC) 4.2% were undiagnosed, and 80% of diagnosed PLWH achieved VLS.[15]

To improve HIV outcomes on a population level, tools and approaches are needed to effectively extend the benefits of HIV treatment to the persons with HIV who to date have not been able to achieve and/or sustain VLS.[16] A disproportionate share of unsuppressed viral load occurs among vulnerable and marginalized populations, as PLWH have elevated rates of mental illness, substance use disorders, and unstable housing,[17–22] which are often co-occurring barriers to achieving desired HIV outcomes. In 2009, the NYC Department of Health and Mental Hygiene (DOHMH) launched the HIV Care Coordination Program (CCP) to support persons at high risk for, or with a recent history of, suboptimal HIV care outcomes.[23] Eligible PLWH include those who are newly diagnosed, have a recent history of poor HIV care outcomes, and/or have documented barriers to care and treatment engagement.[24–26]

A recent effectiveness study demonstrated that the CCP, relative to “usual care,” was effective at promoting VLS at 12 months (VLS 58% versus 52%; RR 1.11, 95% CI 1.08–1.14), especially for newly diagnosed persons (VLS 73% versus 63%; RR 1.15, 95% CI 1.09–1.23) and those who had no evidence of VLS in the year prior to the start of follow-up (VLS 43% versus 32%; RR 1.32, 95% CI 1.23–1.42).[27] The CCP intervention, therefore, may have a vital role to play in efforts to improve health outcomes among PLWH and reduce onward HIV transmission. However, as implemented currently in NYC, the program is expensive, with a per-participant cost of approximately $7,274 in the first year and $5,195 per year in subsequent years. Thus, assessing its cost-effectiveness is a necessary prerequisite for considering scale-up of the program or implementation of the intervention in other settings. The objective of the present study was to use a computer simulation of local HIV progression and transmission to evaluate the cost-effectiveness of the scale-up of the CCP strategy in NYC to all those who appear to be at risk of suboptimal outcomes.

## Methods

A previously validated simulation of HIV progression and transmission[28, 29] in NYC was modified to incorporate the observed effects and costs of the CCP intervention. The simulation estimated the impact and cost-effectiveness of a scale-up of the CCP intervention among all persons at apparent risk for sub-optimal HIV care outcomes in NYC (approximately 35% of PLWH) compared to no implementation of CCP. All study methods were approved by the NYC DOHMH IRB.

### Model overview

The simulation integrates information from an individual-based stochastic Monte Carlo microsimulation of HIV progression with a deterministic compartmental model of HIV transmission.[28, 29] It incorporates downstream health costs potentially saved and infections potentially averted by improved VLS among CCP participants. The simulation is composed of two models. The first model is a natural history model that follows a cohort of HIV-infected patients and predicts time until HIV antiretroviral (ART) failure, accumulation of resistance mutations, and patient death. This progression model provides data to inform the second model, a transmission model. This process is described in detail in S1 Technical Appendix.

**Fig 1:**
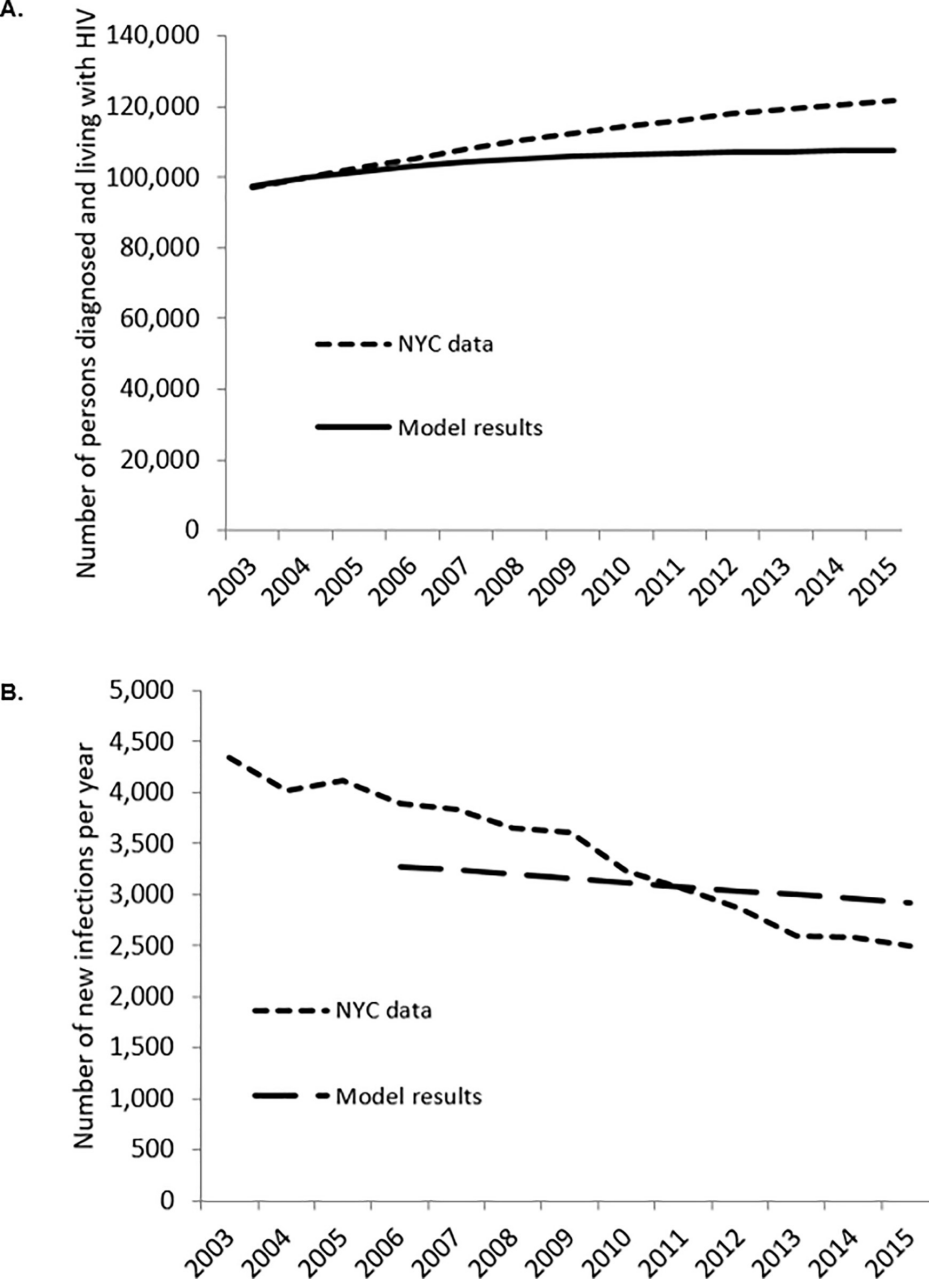
Validation of the HIV epidemic model. a) Comparing model prevalence results with reported data for New York City for 2003–2009. B) Comparing model incidence results with reported data from New York City 2003–2009.

The transmission of HIV is predicted by a compartmental model. Segments of a hypothetical population can become HIV-infected, learn their HIV status, and access and adhere to treatment, which can minimize the risk of onward transmission. Segments of this population can also modify the risk of onward transmission by increasing or decreasing their number of sexual partnerships, condomless sex encounters, exposure to sexually transmitted infections (STIs), and substance use. The model included both sexual transmission of HIV and transmission through syringe and injection-related paraphernalia-sharing during injection drug use (IDU). The probability of transmission between partners was adjusted to account for an infected partner's gender, disease state, and treatment status, and for uninfected partners’ use of pre-exposure prophylaxis (PrEP). The design of the simulation, as well as its calibration and validation (Fig 1), is described in more detail in the S1 Technical Appendix and elsewhere.[28, 29] We used the calibrated simulation to evaluate the impact and value of scaling up the CCP intervention in NYC. Two scenarios were explored. In scenario 1, to represent the practical expectation of approximately two-year average program enrollment periods, the intervention effects and costs were limited to the two years at the start of the simulation. In this scenario the direct benefits of the intervention (i.e. increased VLS) stop at the two-year mark; however, benefits incurred downstream (i.e. transmission events, improvements in life expectancy) are continued after two years. In scenario 2, the program was assumed to be continuously implemented over the full time horizon, with individuals receiving the intervention as they become eligible and remaining in the program until death.

For each scenario, the CCP increased the proportion of PLWH experiencing VLS. The interventions effects were represented in the transmission model by increasing the probability of transitions from “in care but not adherent to ART” to “in care and adherent to ART” compartments. ART adherence was represented as a percentage of pills taken as prescribed per month. ART adherence then influences the likelihood of response to treatment and the achievement of VLS. Since CCP eligibility criteria could not be directly replicated for the model, the scale-up was modeled amongst all HIV-infected individuals who have behaviors associated with risk for nonadherence and/or nonretention in care (i.e., IDU), lessening the likelihood of VLS, and/or higher probability of transmission for a particular VL (e.g., multiple concurrent partners), as surrogate inclusion criteria. A post-hoc sensitivity analysis, “targeted CCP,” aimed to estimate the effectiveness of the intervention if delivered exclusively to the subgroup that had been consistently unsuppressed throughout the year prior to enrollment. However, due to constraints of the simulation architecture, we were unable to limit the intervention’s effect to this specific group. Accordingly, for the “targeted CCP” analysis, we applied the effect size observed in this consistently-unsuppressed subgroup to the same population as in the base case analysis. The simulation was calibrated to NYC epidemiological data, with the goal of replicating trends in NYC HIV prevalence, incidence, and deaths, from 1997 to 2015. The model inputs are described in Table 1.

**Table 1:** Key model input parameters.

Parameter or input	Value	Reference
**Sexual risk characteristics**		
Proportion of population who are abstinent	21.0%	[30]
Probability of monogamous relationship (if sexually active)		
Men who have sex with women (MSW)	78.2%	[31]
Men who have sex with men (MSM)	55.8%	[31]
Women who have sex with men (WSM)	91.1%	[31]
Women who have sex with women (WSW)	48.9%	[31]
Probability of multiple partnerships (if sexually active)		
MSW	21.8%	[31]
MSM	44.2%	[31]
WSM	8.9%	[31]
WSW	51.1%	[31]
Proportion of men who are MSM	5.6%	[31]
Proportion of men who are MSW	94.4%	[31]
Proportion of women who are WSW	2.4%	[31]
Proportion of women who are WSM	97.6%	[31]
**Injection Drug Use Characteristics**		
Proportion of population that injects drugs	1.43%	[32]
Proportion of injection drug users (IDUs) who have unsafe injection practices	32%	[33]
Proportion of IDUs who are male	70%	[33]
**Sexual and IDU transmission**		
Transmission risk per sex act		
Male-to-male	0.00167	[34]
Female-to-male	0.00042	[34]
Male-to-female	0.00081	[34]
Transmission risk per unsafe needle sharing act	0.003	[35]
Relative risk of transmission dependent on viral load	0.16–9.03	[36]
Sex acts (per partnership) per year	89	[37]
Shared injections per year	70	Assumption
**HIV risk behaviors and biological/behavioral modifiers of transmission**		
Prevalence of untreated sexually transmitted infection	6.9%	[38, 39]
Prevalence of unhealthy alcohol use	5%	[40]
Prevalence of consistent condom usage	35%	[31]
**HIV disease related**		
Probability of annual HIV test	31%	[31]
Probability of linkage to care	75%	Unpublished NYC DOMH data
Probability of initiating ART if in care	87%	Unpublished NYC DOMH data
ART adherence	70%	[41]
**Demographics**		
Annual age-related mortality rate	0.0068 (6.8/1000 pop)	[42]
Annual fertility rate	0.0156 (15.6/1000 pop)	[42]
**Intervention**		
RR of VL suppression	RR 1.11	CCP comparison-group study
RR of VL suppression–among not previously suppressed	RR 1.32	CCP comparison-group study
**Costs**		
First-year CCP costs	$7,274	CCP administrative data
Second-year and beyond CCP costs	$5,195	CCP administrative data
Annual cost of care and treatment for individuals with CD4<100	$64,309	[43]
Annual cost of care and treatment for individuals with CD4>100	$33,425	[43]
**Model Population at start of analysis (2015)**		
NYC population size	5,547,672	Model value
Proportion PLWH	2.2%	Model value
CD4 count distribution	<50 cells/mm^3^–7% 50–200 cells/mm^3^–16% 200–350 cells/mm3–27% 350–500 cells/mm^3^–27% >500 cells/mm^3^–23%	Model value
Proportion PLWH with viral suppression	72.2%	Model value
Proportion of PLWH at risk for suboptimal outcomes	40%	Model value
Proportion of PLWH at risk for suboptimal outcomes who were not previously suppressed	41.6%	[27]

Costs and effects were discounted at 3%, and costs were assessed in 2017 US$ from a health sector perspective, which included the cost of treatment. Other than specifying a finite time horizon, all other aspects of the cost-effectiveness analysis were conducted in line with recommendations by the Panel on Cost-Effectiveness in Health and Medicine.[44] The simulated population was HIV-infected and uninfected New Yorkers from the start of the calibration (1997) up through 20 years after the intervention implementation in the year 2015.

### The CCP intervention

The CCP intervention promotes care and treatment engagement among persons at risk for suboptimal HIV outcomes. The CCP combines various evidence-based programmatic elements including case management, patient navigation, directly observed therapy, structured health promotion in home/field visits, and outreach to assist patients in accessing needed medical care and related support services, such as mental health treatment, substance use treatment, and housing assistance. Patients receive and transition through different levels of intensity of programmatic support based on their assessed needs. At the time of this writing, CCP eligibility criteria permitted enrollment of HIV-infected adults or emancipated minors who have residence within the New York grant area with a household income <435% of federal poverty level and who are (1) newly diagnosed with HIV; (2) starting a new ART regimen; (3) never in care or lost to care for at least 9 months; (4) irregularly in care; (5) experiencing ART adherence barriers; or (6) displaying ART resistance or treatment failure. The intervention has previously been described elsewhere.[24, 25] Intervention effectiveness was reported as a relative risk (RR) of the proportion of PLWH experiencing VLS in the CCP versus usual care. The primary outcome incorporated into the HIV model was the proportion of participants experiencing VLS at 12 months and the cost per patient of the intervention (Table 1).

### Cost-effectiveness analysis

We conducted simulations where the CCP intervention was “turned on,” and calculated the health benefits, costs, and cost-effectiveness ratio over the 20-year time horizon. These simulations were compared to a counterfactual scenario representing standard of care in NYC with no CCP intervention. Outcomes measured include total quality-adjusted life years (QALYs) gained, cost per QALY gained, number of new HIV infections averted, and cost per infection averted. As a sensitivity analysis, we varied both intervention efficacy and cost independently across plausible ranges. In cost sensitivity analyses, we maintained the ratio of 1^st^-year costs to costs in the 2^nd^ year and beyond so that the 1^st^-year costs were proportionally higher than all later-year costs throughout the analyses. A cost-per-QALY-gained value less than $100k was considered cost-effective.[45]

## Results

### Effectiveness

At any given time, 42,452 to 51,810 individuals were receiving the intervention. In scenario 1 (2-year average enrollment), scale-up of the CCP intervention reduced the number of new HIV infections over 20 years by 498, versus 33,061 new HIV infections. The number of QALYs gained was 1,055. (Table 2) The number of HIV-related deaths over 20 years was reduced by 62, versus 9,440 deaths.

**Table 2:** Cost-effectiveness by scenario.

	Total Costs (Discounted)	Total Discounted QALYs	New Infections	Cost Change	QALYS Gained (Discounted)	Infections Averted	ICER ($/QALY)	ICER ($/Infection Averted)
	**Time-unlimited enrollment**							
**Standard Care**	$44,131,349,209	104,114,491	33,061	-	-	-	-	-
**CCP**	$47,271,272,410	104,117,837	28,609	$3,139,923,200	4,355	4,453	$720,970	$705,171
	**Time-limited enrollment of two years**							
**Standard Care**	$44,131,349,210	104,114,481	33,061	-	-	-	-	-
**CCP**	$44,578,284,496	104,115,536	32,564	$446,935,286	1,055	498	$423,721	$898,104

**Fig 2:**
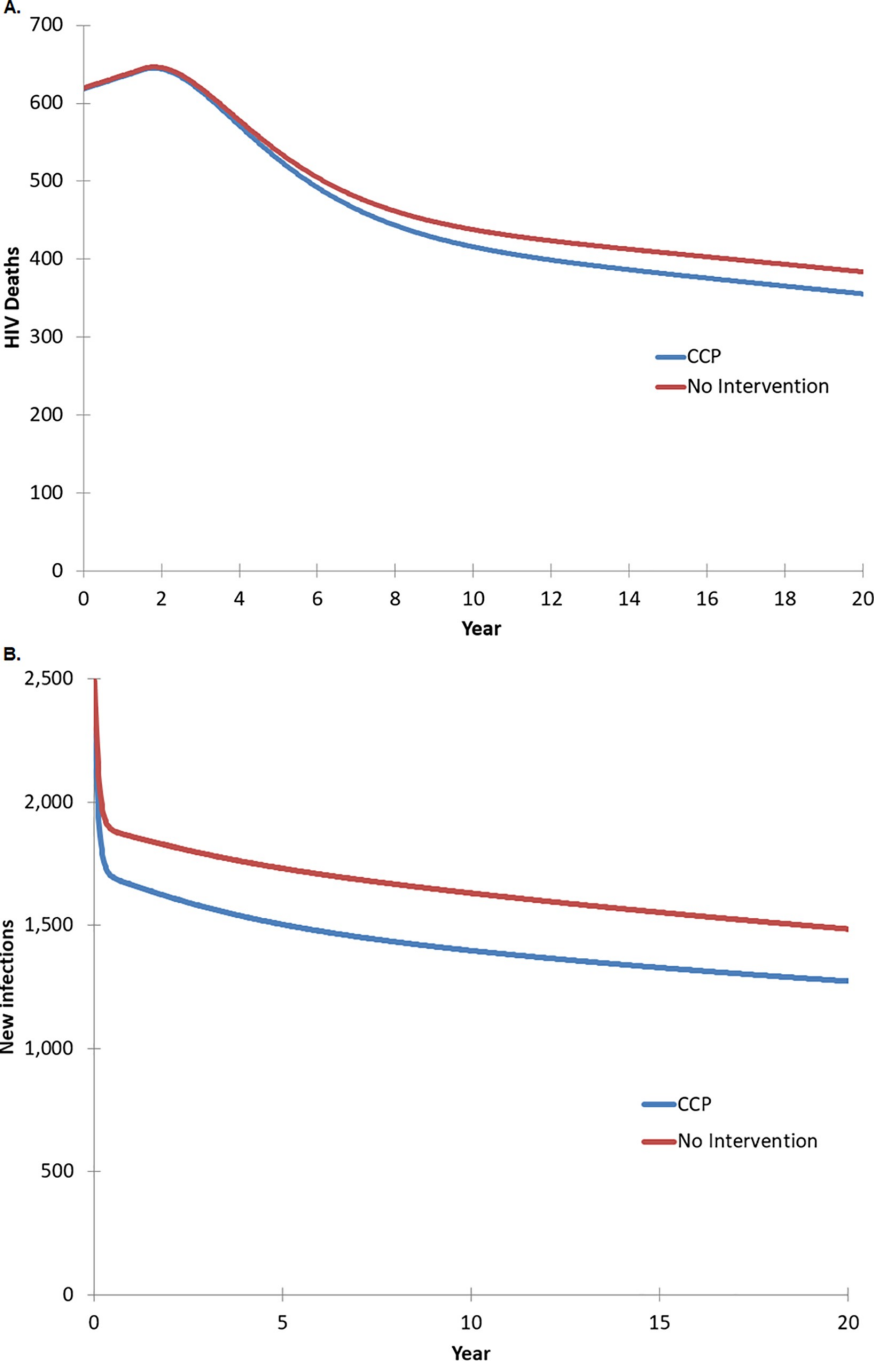
**Impact of continuous CCP implementation on (a) infections averted and (b) HIV related deaths.** Note: The initial sharp drop in new infections is an artifact of the model's run-in period while reaching equilibrium.

In scenario 2 (time-unlimited enrollment), scale-up of the CCP intervention reduced the number of new HIV infections over 20 years by 4,453 infections, versus a base case of 33,061 new HIV infections (Fig 2A). The number of QALYs gained was 4,355. (Table 2) The number of HIV-related deaths over 20 years was reduced by 379, versus 9,440 deaths (Fig 2B).

### Cost effectiveness

In Scenario 1, time-limited enrollment in CCP resulted in a total 20-year discounted cost of $446,935,286, corresponding to costs per infection averted of $898,104. The cost per QALY gained was $423,721. (Table 2)

In Scenario 2, time-unlimited enrollment in CCP resulted in a total 20-year discounted cost of $3,139,923,200, corresponding to costs per infection averted of $705,171. The cost per QALY gained was $720,970. (Table 2)

### Sensitivity analyses

**Fig 3:**
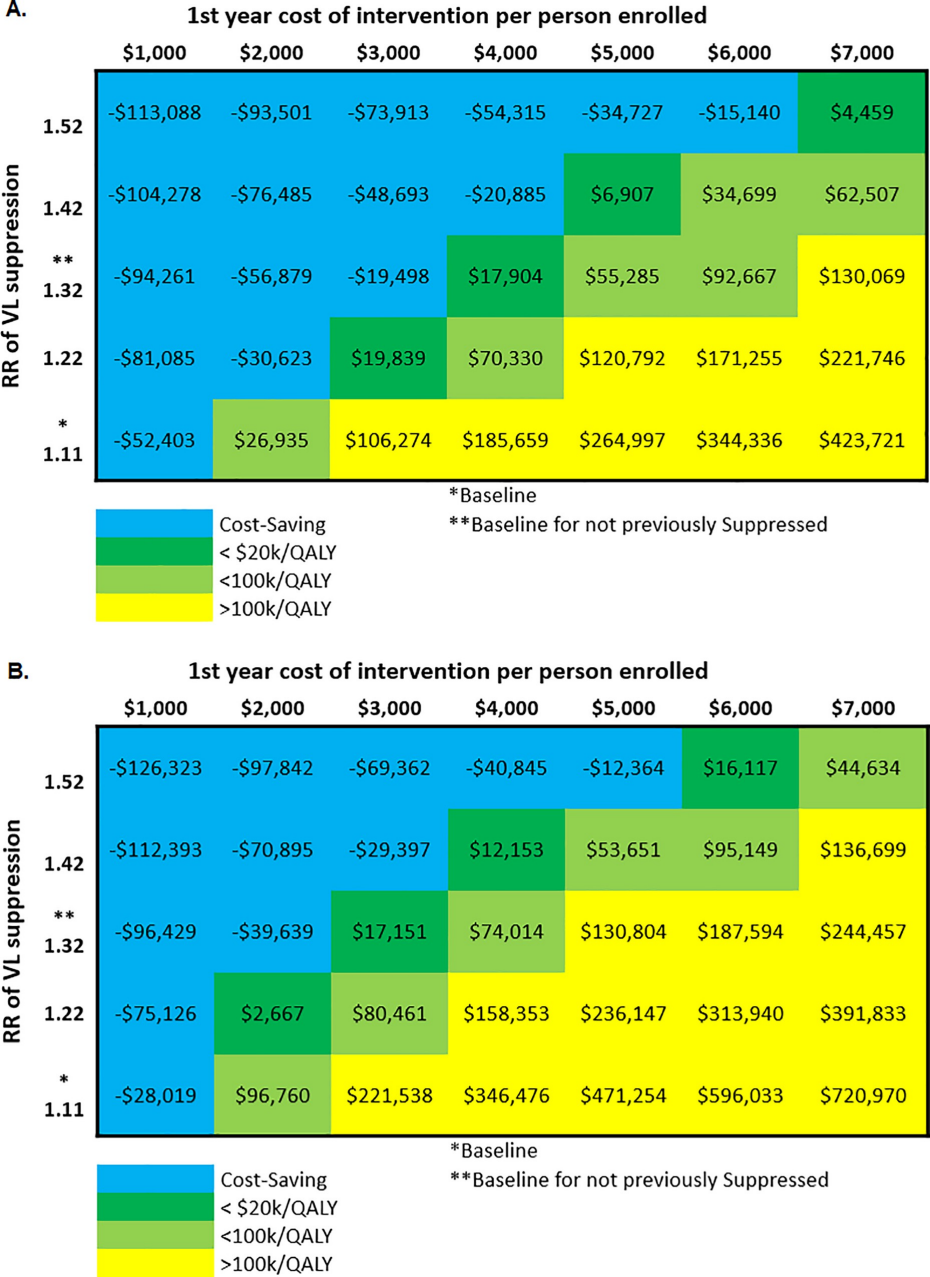
Sensitivity analyses for (a) 2-year implementation and (b) continuous implementation.

The CCP intervention in both scenarios achieved favorable value across lower costs and higher effect sizes (Fig 3A and 3B). When considering a willingness-to-pay threshold of $100,000/QALY, in sensitivity analyses (results not shown) the model outcome was not sensitive to characteristics of the general simulated population (e.g. proportion linked to treatment). However, the model outcome was sensitive to the cost of care and treatment.

If instituted with an average 2-year enrollment time frame (Scenario 1), for the CCP intervention scale-up to become cost effective, the intervention’s effect size must be increased from an RR of 1.11 to an RR of 1.37, or the year-one cost per client must be decreased by more than 41.7%, from $7,274 to $4,241.

If instituted without enrollment time limits (Scenario 2), for the CCP intervention to become cost effective, the intervention’s effect size must be increased from an RR of 1.11 to an RR of 1.47, or the year-one cost per client must be decreased by more than 72.1%, from $7,274 to $2,026, with subsequent annual costs being proportionately decreased.

If instituted with an average 2-year time frame and targeted to persons with unsuppressed viral load in the year prior to enrollment (corresponding to an RR of 1.32), the cost per QALY gained versus no intervention was $130,069. To achieve favorable value, the year-one cost per client must be decreased by more than 14.8%, from $7,274 to $6,196 (Fig 3A). If instituted without enrollment time limits but targeted to persons with unsuppressed viral load (corresponding to an RR of 1.32), the cost per QALY gained was $244,457. To achieve favorable value, the year-one cost per client must be decreased by more than 38.7%, from $7,274 to $4,458, with subsequent annual costs being proportionately decreased (Fig 3B).

Increasing the time horizon to 50 years resulted in an improved cost effectiveness for both implementation scenarios. Scenario 1 resulted in a total 50-year discounted cost of $383,992,076, and 2,043 QALYs gained corresponding to a cost effectiveness of $187,918/QALY. Scenario 2 resulted in a total 50-year discounted cost of $4,202,467,698, and 9,981 QALYs gained corresponding to a cost effectiveness of $237,509/QALY.

## Discussion

We provide estimates of the impact and cost-effectiveness of a hypothetical scale-up of a comprehensive HIV care coordination intervention for promoting VLS among persons in NYC with documented barriers to care and treatment. Our analyses suggest that, from a health sector perspective, a broad scale-up of the CCP was not likely to be cost-effective at current costs and observed levels of effectiveness. Our findings are robust over a range of assumptions regarding cost and effectiveness, with the scaled-up CCP in both the time-unlimited enrollment scenario and the 2-year average enrollment scenario becoming cost-effective only after a decrease in programmatic costs or an increase in effectiveness, while the targeted CCP became cost effective with a smaller decrease in programmatic costs. Thus, CCP scale-up could achieve cost-effectiveness through an increased focus on populations for whom the existing program is most effective, and/or by determining and applying an optimal enrollment period.

In an effectiveness study conducted in a real-world service delivery environment, the CCP intervention was shown to have a significant positive effect on VLS beyond “usual care” in the short-term (i.e., in 12-months of follow-up). Within the study population, a substantial number of individuals (40.1%) were consistently unsuppressed in the year prior to follow-up. This group demonstrated the greatest effect from the CCP compared to the general study population (VLS RR 1.32 vs. RR 1.11).[27, 46] However, our analyses demonstrated that, at current program costs, even in an optimal targeting scenario where the entire population had been consistently unsuppressed during the year before enrollment, the CCP remained above the threshold for cost-effectiveness. Targeting to this population with an enrollment limit of 2 years became cost-effective when programmatic costs were $6,437 in the first year with a proportional reduction in the next year. Consequently, if programmatic costs can be reduced (by 12% from current costs), our models suggest that targeting the CCP for individuals who have been consistently unsuppressed, while applying an average enrollment period of 2 years, may make CCP scale-up a valuable and cost-effective tool in the fight to end the HIV epidemic. The achievement of 90-90-90 goals will depend upon identifying and disseminating such interventions capable of yielding successful HIV treatment outcomes in vulnerable populations.

For the CCP intervention with an enrollment limit of 2 years to be cost-effective in a general high-need population, while maintaining the same programmatic costs, future effectiveness studies must demonstrate a substantial increase in effectiveness (from RR 1.11 to RR 1.46). The CCP effectiveness range is similar to those observed in other interventions; a meta-analysis of similar integrated interventions targeting transmission risk and care continuum outcomes found that the overall effect size on undetectable viral load was OR 1.46 (95% CI 0.93–2.27, p = 0.098).[24] The program’s impact on HIV incidence becomes more prominent over the longer term; specifically, both scenarios become more cost-effective over a 50-year time horizon, reflecting that benefits from secondary infections averted are delayed but important.

Perhaps the most policy-relevant implication of our analyses is that the CCP should be re-conceptualized as a time limited (2-years on average) program for persons with unsuppressed viral load in the year prior to enrollment, and with quality improvement efforts directed at increasing effectiveness from RR 1.32 to RR 1.37 and/or efficiency improvement efforts directed at lowering costs from $7,274 to $6,196 without decreasing effectiveness.

It is also important to note that New York City has a high background rate of VLS attributable to NYC’s robust HIV infrastructure,[14, 47] and therefore the potential benefit of the scaled-up intervention, and its cost-effectiveness, was less favorable than would be likely in other major cities with large HIV epidemics and lower VLS levels. While 85.0% of PLWH were diagnosed and only 57.9% had VLS in 2015 in the US overall,[12, 13] 95.0% of PLWH were diagnosed and approximately 80.0% of diagnosed PLWH had VLS in 2016 in NYC.[15] In addition, NYC has a particularly robust infrastructure for HIV care, such that NYC “usual care” for PLWH includes a variety of other comprehensive care management programs, such as NYS Medicaid “health homes,” the NYS Ryan White Part B-funded Retention and Adherence Program (RAP), supportive HIV housing programs, and the NYS AIDS Drug Assistance Program (ADAP) and ADAP Plus (with health insurance/primary care coverage). The CCP intervention may have a greater additive effect, and therefore more favorable cost-effectiveness, in environments that lack these many complementary programs. Given all of the existing HIV care resources and high background levels of VLS in NYC, the subset of New Yorkers diagnosed with HIV who remain unsuppressed (the final 20%) may also represent a group with particularly complex barriers to treatment engagement, requiring greater effort and cost to resolve in VLS than would be required to achieve VLS among most unsuppressed PLWH in other areas of the US. Similarly, the cost of living in NYC is substantially higher than in other regions and, as a substantial portion of the intervention cost is derived from personnel salary costs, the intervention may also be less costly to implement in other settings outside of NYC. Finally, targeting the program to individuals at highest risk of transmission, as well as at the highest risk of HIV-related complications, may increase the potential for CCP implementation to be cost-effective in NYC, even with current program costs and observed levels of effectiveness.

Multi-level integrated adherence interventions may represent a key strategy to addressing the complex nature of HIV treatment adherence, and its barriers/facilitators.[48–50] As seen in this analysis, however, programmatic costs can greatly impact the cost-effectiveness of the interventions. The multi-component nature of interventions like the CCP also poses a challenge for determining how best to decrease programmatic costs and maximize intervention effectiveness. As a result, in addition to targeting the intervention, further research is needed to determine the most essential components of the CCP intervention and determine if there are components of the intervention that can be scaled back, modified, or eliminated in order to reduce programmatic costs while maintaining program effectiveness. This process is now underway in NYC, through a study comparing the original CCP to a revised CCP model in a stepped-wedge design (R01MH117793).

Our analysis has a number of limitations. First, our model was unable to directly replicate the exact risk strata of patients in the study, and so risks for poor adherence were used as surrogate inclusion criteria in the model. Due to the simulation structure, general high-risk groups (i.e., those with multiple concurrent partners, IDU), each associated with different likelihoods of VLS, are used to represent PLWH without VLS. As a result, the analysis likely overestimates the transmission prevention impact of the program, as increasing VLS among high-risk behavioral groups will have a greater impact on HIV transmission than increasing VLS in groups without (or with lower prevalence of) these behavioral risk factors. Likewise, because the model could not select only consistently unsuppressed individuals for enrollment, the investigation of a targeted intervention utilized the RR1.32 without decreasing the population size, and therefore overestimates the total deaths averted and total QALYs gained. However, costs were also proportionally increased, and thus the cost-effectiveness ratios are likely to be conservative. Second, our model identifies people in broad categories who may be eligible for the CCP, but does not apply actual CCP eligibility criteria.[24–26] This may result in fewer or more people, and different people (i.e., people with fewer or more barriers), being enrolled in the CCP in our simulation of scale-up than may occur in reality.

Third, we conservatively assumed that any treatment effect ceased as soon as the intervention (enrollment) was concluded (after 2 years in Scenario 1 and 20 years in Scenario 2). However, while the program could be implemented over a long period, individuals would be unlikely to receive the intervention and thus incur intervention costs for a full 20 years, and since an intervention effect *has* been observed to endure after intervention termination,[46] this likely underestimates the cost-effectiveness of the program. Fourth, the simulation was calibrated through 2009, the year the CCP began; however, in prospective validation the model under-predicted the number of PLWH in NYC, and this may have resulted in an underestimation of intervention health impact. While the 2015 values do not match exactly, we performed sensitivity analyses around these values and determined that the differences would not change the outcome of the analyses. Fifth, the simulation did not look at the year-by-year uptake of PrEP in NYC. While large-scale PrEP use is likely to reduce the impact of the CCP intervention on the number of new infections averted, PrEP use (although increasing) remains modest in NYC.[51] Therefore, while the cost-effectiveness of the CCP for infections averted may potentially be overestimated, that overestimation is likely to be minimal as CCP specifically targets a low-income, non-white population that is less likely to access PrEP. Fourth, the analysis applied the same intervention costs for all individuals enrolled in the CCP, including those who did not ultimately receive the intervention or dropped out after a short time and those who received the program elements at varying levels of intensity. This likely overestimates the total cost of implementation. Finally, our simulation does not measure additional programmatic effects on social determinants of health such as housing, engagement in mental health care, substance use treatment, or treatment for comorbidities, which likely impact both health and quality of life. Consequently, the total health impact of the intervention on QALYs is potentially underestimated.

## Conclusion

Our results suggest that CCP implementation within NYC would require a more targeted approach and reduced costs to achieve cost-effectiveness. However, the CCP with a 2-year average enrollment period has the potential to be cost-effective in environments with a lower background of VLS, and/or when restricted to those who have been consistently unsuppressed in the year prior to CCP enrollment, and/or where substantial reductions in programmatic costs could be achieved.

## Supporting information

S1 Technical Appendix Supplementary methods. (DOCX)
